# Lung pericytes as mediators of inflammation

**DOI:** 10.1152/ajplung.00354.2022

**Published:** 2023-05-02

**Authors:** Samuel G. Rayner, Chi F. Hung, W. Conrad Liles, William A. Altemeier

**Affiliations:** ^1^Division of Pulmonary, Sleep, and Critical Care Medicine, Department of Medicine, University of Washington, Seattle, Washington, United States; ^2^Center for Lung Biology, University of Washington, Seattle, Washington, United States; ^3^Division of Allergy and Infectious Diseases, Department of Medicine, University of Washington, Seattle, Washington, United States

**Keywords:** ARDS, endothelial cells, inflammation, pericyte, sepsis

## Abstract

Pericytes are microvascular mural cells that directly contact endothelial cells. They have long been recognized for their roles in vascular development and homeostasis, but more recently have been identified as key mediators of the host response to injury. In this context, pericytes possess a surprising degree of cellular plasticity, behaving dynamically when activated and potentially participating in a range of divergent host responses to injury. Although there has been much interest in the role of pericytes in fibrosis and tissue repair, their involvement in the initial inflammatory process has been understudied and is increasingly appreciated. Pericytes mediate inflammation through leukocyte trafficking and cytokine signaling, respond to pathogen-associated molecular patterns and tissue damage-associated molecular patterns, and may drive vascular inflammation during human SARS-CoV-2 infection. In this review, we highlight the inflammatory phenotype of activated pericytes during organ injury, with an emphasis on novel findings relevant to pulmonary pathophysiology.

## Graphical abstract

**Figure:**
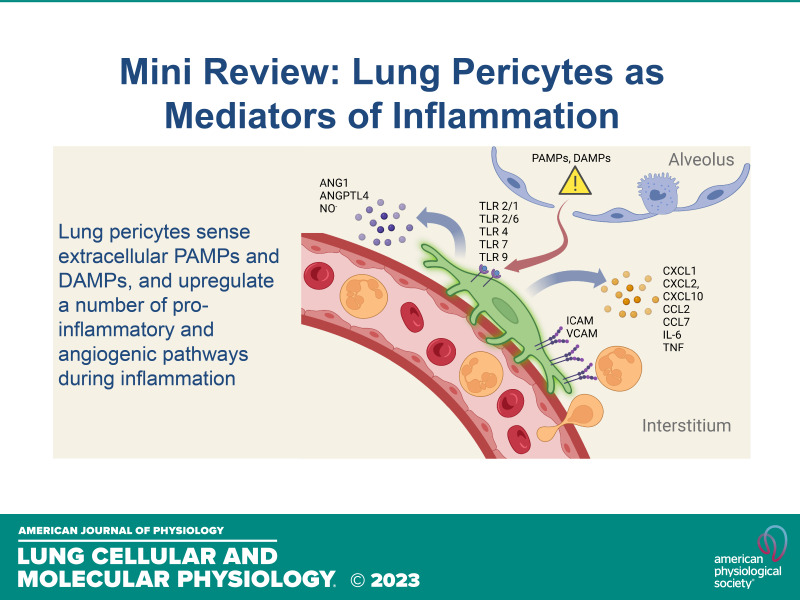

## INTRODUCTION

Pericytes are integral to the microvascular unit during development and in homeostasis (1). Pericytes and endothelial cells contribute to a shared basement membrane. The basement membrane separating pericytes and endothelial cells is not continuous, allowing “peg-and-socket” physical contacts between pericytes and endothelial cells to develop in these gaps in the basement membrane (1). Direct cell-cell communication is thought to occur in these gaps. In addition, as each pericyte borders more than one endothelial cell, it may be able to integrate communication from several endothelial cells. This anatomic relationship allows rapid bidirectional signaling between endothelial cells and pericytes.

Originally described in human lung capillaries by Weibel (2) in 1974, characterization of pericytes required ultrastructural localization by electron microscopy (EM). In the past two decades, investigators have been interested in pericytes as more than mere structural cells in the quiescent microvasculature. Characterizing pericytes by EM, however, is difficult to accomplish on a routine basis in laboratory investigation. To facilitate isolation and examination of pericytes, other methods to characterize pericytes or pericyte-like cells were developed. These include a combination of markers commonly expressed by pericytes and histology demonstrating pericyte morphology and juxtaposition to endothelial cells. However, no single molecular marker is specific for pericytes. Moreover, pericyte markers may be organ- and species-specific, and marker expression may be dynamic during development and injury. Common markers used in the literature to identify pericyte-enriched populations are listed in Table 1 (3–11, 17–25).

**Table 1.** Common markers used in the literature to identify pericyte-enriched populations

Human	Organ
PDGFRβ (3–8)	CNS, retina, lung, heart, kidney, skin
Chondroitin sulfate proteoglycan 4 (NG2) (3, 5–7)	CNS, retina, lung, heart
Desmin (3, 7)	CNS, retina
Smooth muscle actin, α (3, 7)	CNS, retina
RGS5 (9)	CNS
CD13 (3, 7)	CNS, retina
CD146 (3, 5, 10)	CNS, lung
Mouse	
PDGFRβ (4, 11–16)	CNS, lung, heart, kidney, skin
NG2 (6, 13, 14, 17)	CNS, lung, heart
Gli1 (18, 19)	Kidney, lung, heart, liver
CD13 (20, 21)	CNS
CD146 (22, 23)	CNS, lung
RGS5 (9, 24, 25)	CNS

CNS, central nervous system.

In the healthy state, the microvascular unit is characterized by a state of quiescence. Endothelial cells form tight junctions with adjacent endothelial cells, express low levels of inflammatory markers and leukocyte adhesion molecules, exhibit anticoagulative properties, and in the lung, high nitric oxide synthesis to maintain vasodilation (26). Signaling between pericytes and endothelial cells during homeostasis encourages an antiangiogenic and anti-inflammatory endothelial cell phenotype, including paracrine signals such as angiopoietin-1, platelet-derived growth factor B (PDGFB), vascular endothelial growth factor (VEGF), and transforming growth factor-β (TGF-β) (27–29).

With injury, the microvasculature becomes “activated” with a series of physiological events: inflammatory cell recruitment, vascular leakage, coagulopathy, and disruption to nitric oxide synthesis or responsiveness. Investigation of these events has traditionally focused on the role of endothelial cells. In recent years, however, it has become increasingly recognized that the subendothelial layer of the microvascular unit, marked by pericytes and extracellular matrices, also plays crucial roles in inflammation and repair.

Seminal studies of pericytes as progenitors of scar-forming cells in the skin, central nervous system (CNS), kidneys, and lungs positioned this stromal subtype as a dynamic population in fibrosis (12, 13, 30–33). Although focus on pericytes as mediators of repair has contributed significantly to the scientific debate on the cellular basis of fibrosis, there is also increasing evidence in the literature to suggest pericytes as functional mediators of inflammation during acute injury.

**Figure 1.**
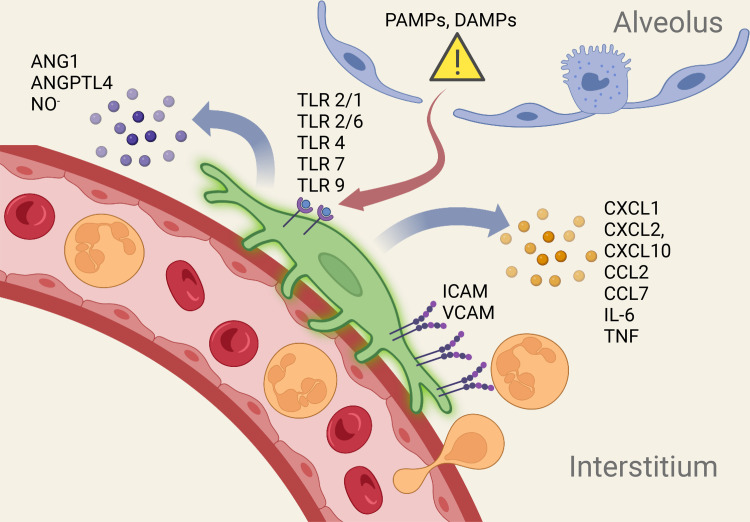
DAMPs and PAMPs gain entry into lung interstitium through disrupted alveolar epithelial barrier to activate PRRs on periyctes. Activated lung pericytes express cytokines, chemokines, adhesion molecules, and angioactive mediators to coordinate a local inflammatory response. ANG1, angiopoietin-1; ANGPTL4, angiopoietin-like 4; DAMPs, damage-associated molecular patterns; ICAM, intercellular adhesion molecule; NO, nitric oxide; PAMPs, pathogen-associated molecular patterns; PRRs, pattern recognition receptors; TLR4, Toll-like receptor-4; TNF, tumor necrosis factor; VCAM, vascular cell adhesion molecule. [Image created with BioRender.com and published with permission.]

In this review, we focus on the mechanisms by which pericytes activate local inflammatory responses after lung injury. Work from our group, using transgenic models that label mouse lung pericytes, has demonstrated robust inflammatory responses in pericytes and attenuated lung inflammation when pericytes are selectively depleted (14–16, 34). Although this review focuses on the role of perivascular cells in lung inflammation, studies in extrapulmonary organs, such as the CNS and the heart, reveal similar biological responses to organ injury (35, 36). Recent literature on pathogen and damage pattern recognition, cytokine/chemokine secretion by pericytes, leukocyte trafficking, and pericyte-endothelial cross talk will be highlighted to illustrate the multiple potential roles of pericytes in inflammation (Fig. 1).

## RECRUITMENT AND TRAFFICKING OF LEUKOCYTES

An important component of the initial inflammatory response to tissue injury in the lung and other organs is recruitment of circulating leukocytes. In the alveolar compartment of the lung, leukocytes transmigrate across alveolar capillary walls following luminal interaction with endothelial adhesion molecules such as intercellular adhesion molecule 1 (ICAM-1) and vascular cell adhesion molecule 1 (VCAM-1) (37). After crossing the endothelium, leukocytes migrate across the capillary basement membrane and interstitium in response to chemokine gradients and access the intra-alveolar space through epithelial transmigration (37). Tissue-resident innate immune cells, such as macrophages and dendritic cells, are traditionally thought to be the sentinels that regulate endothelial activation and chemokine-directed leukocyte trafficking. However, recent data in both extrapulmonary tissue and the lung implicate pericytes as potential sentinel cells, communicating directly with endothelial cells on the abluminal side of the microvasculature following injury.

Activated pericytes induce adhesion molecule expression in endothelial cells (38, 39). Two studies, using intravital microscopy in cremaster muscle and skin preps, respectively, have shown that pericytes, in an ICAM-1-dependent manner, regulate interstitial migration of leukocytes following transmigration across the vascular wall (40, 41). These studies implicate pericytes as potential regulators of leukocyte trafficking following inflammatory activation in nonpulmonary tissue.

In addition to leukocyte adhesion, pericytes also facilitate leukocyte trafficking through cytokine release. Like other stromal cells, pericytes are capable of sensing damage-associated and pathogen-associated molecular patterns [damage-associated molecular patterns (DAMPs) and pathogen-associated molecular patterns (PAMPs), respectively)]. Pericytes in multiple organs express functional pattern recognition receptors (PRRs), such as Toll-like receptors (TLRs) and NOD-like receptors (NLRs). Human neurovascular pericytes, for instance, express TLR 2, 4, 5, 6, and 10 and NOD1, NOD 2, NLRPs 1–3, 5, 9, 10, and NLRX at the transcript level in vitro. Activation of select PRRs in cultured human neurovascular pericytes leads to inflammasome formation and secretion of inflammasome-cleaved pro-IL-1β (42). In response to DAMPs and PAMPs, pericytes become “activated” and secrete a host of proinflammatory cytokines (25, 43–48). In the central nervous system (CNS), for example, human brain pericytes secrete CCL2, CCL5, CXCL1, CXCL10, CXCL11, and IL-8 in response to lipopolysaccharide (LPS) as well as cytokines such as TNF and IL-1β. Murine pericytes isolated from the brain exhibit a similar response to LPS stimulation (21). In the retina, TNF and IL-1β stimulation of human retinal pericytes induces the production of CCL2, CCL3, CCL11, G-CSF, and IL-8.

Work by our group and by our collaborators demonstrate a similar proinflammatory secretome in both murine and human lung pericytes (Table 2). Pericytes in the lung reside in the perivascular niche under homeostasis and are protected from exposure to alveolar contents. However, when there is a breach in the alveolar epithelial barrier, contents from the alveolar space during injury may activate quiescent lung pericytes into an inflammatory phenotype via pattern recognition receptor (PRR) signaling. We demonstrated that primary mouse lung pericytes express functional TLRs 2/1, 2/6, 4, 7, 9 (15). In response to TLR agonists, lung pericytes upregulate cytokine expression, including chemokines for neutrophils, monocytes and lymphocytes, and adhesion molecule expression in vitro and in vivo (15, 16). Furthermore, cultured mouse lung pericytes exposed to alveolar contents from uninjured and sterilely injured lungs [bronchoalveolar lavage fluid (BALF) collected from uninjured and injured mice] induced inflammatory programs in lung pericytes (15). Cultured human lung pericyte-like cells, defined by PDGFRβ, behave similarly to mouse lung pericytes and adopt a proinflammatory phenotype in response to PRR signaling, suggesting a role in leukocyte recruitment in the lung (49). RNAseq analysis of global transcriptional changes in activated mouse lung pericytes revealed inflammatory programs as the most highly upregulated biological processes, confirming the proinflammatory phenotype previously demonstrated using TLR agonists and BALF from injured mice (16).

**Table 2.** Pattern recognition receptor signaling in lung pericytes

Signaling	Species	Cytokines, Chemokines, and Adhesion Molecules
TLR 2/1	Mice	*Cxcl1, Cxcl2, Cxcl10, Ccl2, Tnf, Icam*
TLR 2/6	Mice	*Cxcl1, Cxcl2, Cxcl10, Ccl2, Tnf, Icam*
TLR 4	Mice	*Cxcl1, Cxcl2, Cxcl10, Ccl2, Ccl7, IL-6, Tnf, Icam*
TLR 7	Mice	*Cxcl1, Cxcl2, Cxcl10, Ccl2, Tnf, Icam*
TLR 9	Mice	*Cxcl2, Tnf*
Injured BALF (DAMP)	Mice	*Cxcl1, Cxcl2, IL-6, Ccl2, Ccl7, Ccl20, Ifng, Nos2, Vcam1*
TLR 4	Human	*Cxcl1, Cxcl8, Ccl2*
TLR 2/6	Human	*Cxcl1, Cxcl8, Ccl2*
Necrotic cell lysate (DAMP)	Human	*Cxcl1, Cxcl8, Ccl2*

BALF, bronchoalveolar lavage fluid; DAMPs, damage-associated molecular patterns; PAMPs, pathogen-associated molecular patterns; TLR, Toll-like receptor.

In vitro data from recently published work suggest that the CCR2-CCL2/CCL7 chemotaxis pathway may play a functional role in the recruitment of CCR2^+^ monocytes to the lung during lung injury. We showed that conditioned media from activated pericytes enhance chemotactic migration of bone marrow-derived macrophages (BMDMs), which is reversed with CCR2 blockade (receptor for CCL2 and CCL7) using a small molecular antagonist (16). Future studies are needed to further delineate the functional consequence of pericyte-dependent leukocyte recruitment in response to pathogen clearance, lung injury, and resolution of tissue damage.

## PERICYTE-ENDOTHELIAL CELL INTERACTIONS IN ACUTE AND CHRONIC INFLAMMATION

### Acute Inflammation

In addition to recruiting leukocytes, pericytes may modulate endothelial function during acute injury. Evidence suggests that pericyte/endothelial cross talk is crucial in states of acute systemic inflammation such as sepsis (1, 50, 51). The angiopoietin family of proteins is especially important in this context. Angiopoietins-1 and -2 (ANG1 and ANG2) participate in pericyte-endothelial signaling in both health and disease through their effects on the Tie family of receptor tyrosine kinases. TIE1 and TEK/TIE2 receptors are found principally on endothelial cells (52, 53). TEK acts as a receptor for ANG proteins and forms a heterodimer with TIE1 following ANG binding, leading to downstream signaling. ANG1 is a strong agonist for TEK, and in states of quiescence, ANG1 is released by mural cells including pericytes, leading to TEK phosphorylation and downstream signaling that promotes vascular stability and decreased permeability (54). Among other effects, TEK signaling strengthens barrier function by enhancing accumulation of the protein VE-cadherin at intercellular adherens junctions and reduces leukocyte adhesion and inflammation through reductions in vascular cell adhesion molecule (VCAM), E-selectin, and intercellular adhesion molecule-1 (ICAM-1) expression (55).

ANG2 is produced by endothelial cells and stored in specialized organelles known as Weibel-Palade bodies (55). These organelles allow rapid release of ANG2 in response to endothelial activation, leading to autocrine signaling on endothelial cells (56). ANG2 exerts context-specific effects on the TEK receptor (57). In contrast to ANG1, ANG2 antagonizes TEK signaling during inflammation, resulting in increased vessel permeability, increased angiogenesis, and vessel destabilization with loss of pericyte coverage. Interestingly, although TEK is classically associated with endothelial cells, low levels of the receptor were recently found to be expressed on pericytes, with apparent functional effects, highlighting the complicated and bidirectional nature of pericyte/endothelial signaling (58). Mice that overexpress ANG2 developed pericyte loss, increased vascular permeability, and cardiovascular alterations (53, 59). Pericyte recruitment through PDGFB was able to reverse these changes, suggesting that ANG2 signaling in pericytes may contribute to vascular pathology in septic shock.

Another angiogenic product that is upregulated in activated lung pericytes is angiopoietin-like 4 (ANGPTL4). The 50 kDa full-length protein is secreted and cleaved into C-terminus and terminus products by furin and other proteases in the extracellular space (60). In cancer biology and studies of retinopathies, the c-ANGPTL4 fragment has been shown to suppress intercellular tight junction proteins in endothelial cell lines and enhance microvascular permeability (61). Our laboratory has shown that activated lung pericytes highly express *Angptl4* and conditioned media from activated lung pericytes reveal high levels of ANGPTL4 by ELISA (16). Moreover, knockdown of *Angptl4* by siRNA in activated lung pericytes resulted in conditioned media with attenuated angiogenic potential compared with conditioned media from activated lung pericytes without *Angptl4* knockdown (16). These findings in activated pericytes suggest potential mechanisms of pericyte-endothelial cross talk during acute lung injury. Investigations into the functional relevance of these pathways in pericyte-endothelial cross talk are ongoing.

In addition to angiopoietins, lung pericytes may regulate endothelial biology through other pathways during acute inflammation. We recently showed that activated lung pericytes upregulate mediators that alter microvascular biology (16). For example, *Nos2* is significantly upregulated in activated lung pericytes and leads to increased nitrites (a stable breakdown product of nitric oxide) in conditioned media of activated lung pericytes. NOS2 is an inducible nitric oxide synthase that metabolizes l-arginine to nitric oxide. Nitric oxide is a well-recognized regulator of endothelial cell proliferation and microvascular tone (62). Recent evidence also suggests that nitric oxide generated through the NOS2 pathway enhanced angiogenic airway remodeling and inflammation in the asthma model (63). Given the intimate anatomic relationship between lung pericytes and microvascular endothelial cells, pericyte-specific NOS2 expression may have played a functional role in driving endothelial activation in injury and warrants further investigation.

### Chronic Inflammation

Pericyte regulation of endothelial biology may be a crucial axis in chronic inflammation. Experimental evidence in extrapulmonary organs suggests pericytes may be necessary to maintain endothelial cell quiescence. Pericyte-deficient mice show increased brain endothelial expression of leukocyte adhesion molecules (64). Recently, it was shown that increased endothelial vascular cell adhesion molecule 1 (VCAM-1) and intercellular adhesion molecule 1 (ICAM-1) in pericyte-depleted mice lead to increased brain leukocyte infiltration and worse outcomes in experimental CNS autoimmune disease (65). Studies are needed to assess whether disrupted pericyte-endothelial signaling during human inflammatory disease leads to increased leukocyte trafficking in this manner.

Pericyte regulation of endothelial cells in chronic human inflammatory disease has been best studied in diabetic retinopathy. Diabetic retinopathy progresses through an initial nonproliferative phase characterized by retinal vascular abnormalities including microaneurysms, microhemorrhages, nerve infarcts, and retinal edema, before entering a stage of proliferative retinopathy with dysregulated endothelial sprouting and high vascular permeability (66). Hyperglycemia-induced inflammation plays a critical role in diabetic retinopathy and may drive changes in vasoregression and subsequent neovascularization (67, 68). Pericyte loss is one of the earliest findings in diabetic retinopathy and contributes to vascular instability (67, 69). Intriguingly, significant Ang2 upregulation precedes pericyte loss in diabetic retinopathy, possibly due to increased endothelial production of Ang2 in the setting of hyperglycemia (67, 70, 71). Supporting the idea that pericyte loss may drive inflammation in diabetic retinopathy, decreased pericyte recruitment through endothelial PDGFB knockout led to abnormalities similar to those seen in diabetic retinopathy, and inhibition of retinal pericyte recruitment in a murine postnatal angiogenesis model resulted in endothelial inflammatory responses and breakdown of the blood-retina barrier (72, 73). Similar responses are seen elsewhere in the central nervous system, where depletion of pericytes results in increased vascular permeability and heightened inflammatory responses (64, 65).

## DEFINING A FUNCTIONAL ROLE IN INFLAMMATION THROUGH PERICYTE ABLATION

Although evidence in the literature suggests pericytes may contribute to tissue inflammation by trafficking and attracting leukocytes, or by signaling to the endothelium, there remains scant evidence demonstrating functional contribution by pericytes. To study this question, our group investigated the functional relevance of pericytes by examining the effect of pericyte loss on lung inflammation. We developed a model for pericyte ablation in the lung by administering diphtheria toxin (DT) to transgenic mice with pericyte-restricted simian DT receptor (iDTR) expression (14). Direct instillation of DT in the lungs caused moderate inflammation in mice, as evidenced by elevated white blood cell (WBC) counts and total protein in the bronchoalveolar lavage fluid (BALF). In DT-sensitive mice, direct instillation of DT reduces PDGFRβ+ cells by 40–50%, as assessed by flow cytometry. Interestingly, although DT-insensitive mice show signs of moderate lung inflammation from DT exposure, pericyte ablation in DT-sensitive mice significantly decreased inflammation and protein in the alveolar space. This effect may be due to dampened cytokine/chemokine secretion and leukocyte trafficking in pericyte-ablated mice. Furthermore, pericyte ablation in the bleomycin-induced lung injury model also reduced inflammation in the acute inflammatory phase at *day 3* (14) and *day 7* (34), confirming a functional role for lung pericytes in sterile injury.

It is important to note, however, that these findings reflect only inflammation in the acute phase as the effect of DT ablation is only transient. Twenty-eight days after single DT administration, the level of PDGFRβ+ stromal cells in DT-sensitive mice returned to a level comparable to that of DT-insensitive mice. Therefore, the effect of long-term pericyte loss on measures of inflammation and endothelial barrier function remains uncharacterized and will require further study using a chronic model of lung pericyte ablation.

## RELEVANCE IN THE ERA OF SARS-CoV-2/COVID-19

Overexuberant microvascular activation leading to excessive systemic inflammation and capillary leak likely plays a role in the pathogenesis of severe COVID-19, the human disease caused by SARS-CoV-2 infection (74). Although initial reports focused on endothelial cell dysfunction in COVID-19, a recent study demonstrated that perivascular mural cells play a central role in the pathogenesis of severe COVID-19 (75). The authors evaluated expression of the angiotensin-converting enzyme-2 (ACE2) receptor, to which the SARS-CoV-2 virus binds, in multiple organs from adult and developing mice. They found that microvascular ACE2 expression occurred in pericytes, and not endothelial cells as previously suspected. Supporting this concept, SARS-CoV-2 infects human cardiac pericytes in vitro, upregulating vasoactive signaling and causing pericyte death (76). Human autopsy studies on patients with COVID-19 also reveal diffuse perivascular cell loss (77), which is important as pericyte deficiency in mice has been shown to promote endothelial activation, altered arterio-venous molecular signatures in endothelial cells, and disruption of the CNS blood-brain barrier (78).

Taken together, the published reports raise the intriguing possibility that viral-induced activation or pericyte loss may drive the endothelial dysfunction seen in severe COVID-19 infection. Whether inflammatory or vasoplegic signaling from activated pericytes or pericyte rarefraction due to cell death may be driving vascular pathology in severe COVID infection requires further study. Better understanding the tropism of SARS-CoV-2 for pericytes and the effect of viral infection on pericyte biology may reveal important mechanistic insights into the microvascular dysfunction and hyperinflammation observed in severe COVID-19 infections.

## CONCLUSIONS

There are important considerations when examining the literature on pericyte biology. Although pericytes from different organs exhibit similarities in biological response during acute injury, the physiological niche of each organ is unique and pericyte response is likely context specific. Unlike pericytes in the systemic circulation, pericytes in the pulmonary vasculature receive the entire cardiac output from the right heart, with oxygen tensions, shear forces, and cyclic stretch from respiration that are unique to the lung microvascular niche. Although the biology of pericytes across organs may exhibit overlap in their proinflammatory and reparative pathways, differences also likely exist and findings from extrapulmonary pericytes require validation in the lung.

Another important consideration is the absence of a truly pericyte-specific marker. Markers that define pericyte-enriched populations are also expressed in other mesenchymal populations such as vascular smooth muscle cells and fibroblast subtypes. Moreover, expression of these markers may be dynamic with organ injury. For example, PDGFRβ is often used as a marker that defines stromal cells enriched for pericytes. However, studies have revealed that stromal cells that are PDGFRβ^−^ may express PDGFRβ de novo under stress. PDGFRβ^−^ vascular smooth muscle cells, for instance, express PDGFRβ during inflammation in atherosclerotic lesions, rejected transplanted kidneys, chronic synovitis, and peri-infarcts in the brain (79). Human skin fibroblasts, which are PDGFRβ^−^ in situ, become PDGFRβ^+^ as they are passaged in vitro (80). Finding reliable markers that faithfully trace pericytes in different organs will be an important step toward better understanding of these cells in organ injury. Many groups have leveraged advances in transgenic animal models to lineage-trace pericyte populations using genetic fate mapping (81). Our own group has experience with using the *Foxd1* promoter in mice to lineage-trace pericytes of mesenchymal origin in bleomycin-induced lung injury (13). With advances in single-cell technologies, identification of pericytes and their subtypes by transcriptional or proteomic signatures may further advance our identification and understanding of these cells.

Although evidence from our group illustrates potential inflammatory roles for pericytes in the lung, the precise mechanisms by which lung pericytes mediate local inflammation and alter endothelial biology have yet to be established. It is not known whether all pericytes are proinflammatory. Pericytes are capable of numerous biological responses beyond inflammation, including angiogenesis, fibrosis, and cellular regeneration. Whether pericytes are functionally plastic or whether subsets of pericytes are fated for inflammatory, angiogenic, fibrogenic, or regenerative programs upon activation remain open questions.

Both lung injury and repair are orchestrated by diverse cell types in the lung. Contribution by stromal cells, or “interstitial cells,” remains an area that is often overlooked. Owing to advances in molecular technologies, we are now able to characterize this heterogenous class of cells at a more detailed and granular level. Evolving understanding of paracrine signaling pathways in pericyte-endothelial interaction has unveiled novel biological functions of pericytes during organ injury. Further work in this area will fill in important gaps in our understanding of lung injury and repair, with novel cellular and molecular insights that may offer novel targets for therapeutic intervention.

## GRANTS

This work was supported by funding from the National Institutes of Health (NIH) R03HL155075 and R01HL166273-01 (to C.F.H.) and Institutional KL2 training award 5KL2TR002317-04 (to S.G.R.).

## DISCLOSURES

No conflicts of interest, financial or otherwise, are declared by the authors.

## AUTHOR CONTRIBUTIONS

S.G.R. and C.F.H. prepared figures; S.G.R. and C.F.H. drafted manuscript; S.G.R., C.F.H., W.C.L., and W.A.A. edited and revised manuscript; S.G.R., C.F.H., W.C.L., and W.A.A. approved final version of manuscript.
